# Editorial: The Immunomodulatory Properties of Extracellular Vesicles From Pathogens, Immune Cells, and Non-immune Cells

**DOI:** 10.3389/fimmu.2018.03024

**Published:** 2018-12-19

**Authors:** Ivan K. H. Poon, Christopher D. Gregory, Maria Kaparakis-Liaskos

**Affiliations:** ^1^Department of Biochemistry and Genetics, La Trobe Institute for Molecular Science, La Trobe University, Melbourne, VIC, Australia; ^2^Research Centre for Extracellular Vesicles, School of Molecular Sciences, La Trobe University, Melbourne, VIC, Australia; ^3^The Queen's Medical Research Institute, University of Edinburgh Centre for Inflammation Research, Edinburgh, United Kingdom; ^4^Department of Physiology, Anatomy and Microbiology, School of Life Sciences, La Trobe University, Melbourne, VIC, Australia

**Keywords:** apoptotic cells, apoptotic bodies, bacterial membrane vesicles, exosomes, extracellular vesicles, immunomodulatory, microvesicles, tumor cells

## Introduction

Intercellular communication is key for immune regulation and extracellular vesicles (EVs) are emerging as important mediators of this process. EVs like exosomes, microvesicles, and apoptotic bodies are membrane-bound vesicles that can be released by both immune and non-immune cells. Although different types of EVs vary greatly in their size (~30 nm to 5 μm in diameter) and mechanism of formation, it is now well-established that the cellular constituents in/on EVs (e.g., antigens, cytokines, membrane proteins, and microRNAs) can regulate a variety of immune responses. Besides mammalian cells, bacteria, fungi, and parasites can also release membrane vesicles to modulate host immune responses. In this research topic, a collection of primary research and review papers explored the immunoregulatory properties of EVs released from immune cells, tumor cells, apoptotic cells as well as pathogens.

## Immunoregulatory Properties of EVs Released From Granulocytes and Macrophages

EVs can be released from a variety of cell types, in particular by immune cells to regulate immune responses (1). In this research topic, small and large EVs (<220 nm and >220 nm, respectively) generated from granulocytes were described by Danesh et al. to exhibit immunostimulatory properties on monocytic cells. Interestingly, the authors also observed a positive association between the levels of CD66b^+^ granulocyte-derived EVs with mortality in intensive care unit patients. In another study in this research topic, Alvarez-Jimenez et al. described the ability of EVs generated from *Mycobacterium tuberculosis*-infected neutrophils (~100–700 nm) to promote the release of proinflammatory cytokines from macrophages and removal of intracellular *M. tuberculosis* via an autophagy-dependent mechanism. Furthermore, Singhto et al. examined the role of macrophage-derived EVs (~50–80 nm) in the context of kidney stone pathogenesis. The authors described the proteomic profile of macrophage-derived EVs following calcium oxalate monohydrate crystals (a type of crystal that is more pathogenic in kidney stone disease) treatment, and how these EVs could modulate a variety of immune cell functions *in vitro*. Collectively, these studies highlight the immunomodulatory properties of EVs generated from immune cells.

## Immunoregulatory Properties of EVs Released From Tumour and Apoptotic Cells

Tumor cells can play a key role in establishing a microenvironment that favors their growth, and a variety of soluble factors released from tumor cells including VEGF and IL-10 have been shown to facilitate this process (2). Similarly, tumor cell-derived exosomes and microvesicles have also been reported to contribute to the establishment of tumor microenvironment (3, 4). In this research topic, Dörsam et al. reported the ability of EVs (~130 nm) generated from Hodgkin lymphoma to promote recipient fibroblasts to exhibit a cancer-associated fibroblast phenotype, resulting in the release of pro-inflammatory cytokines, growth factors and pro-angiogenic factors that could facilitate a tumor supportive environment. In another research article, Dionisi et al. explored a different concept and demonstrated the ability of tumor (Burkitt's lymphoma)-derived microvesicles (3 predominant EV populations of ~105, 175, and 285 nm) carrying tumor antigens to enhance cross-processing ability of clinical grade dendritic cells and facilitate activation of CD8^+^ T cells. These findings suggest the potential use of tumor cell-derived microvesicles to promote the efficacy of dendritic cell-based vaccines for anti-tumor immunotherapy.

In addition to EVs released from healthy/viable tumor cells, two reviews by Gregory and Dransfield and Muhsin-Sharafaldine and McLellan discussed the recent literature on the ability EVs released from apoptotic tumor cells to modulate tumor growth and anti-tumor immunity. Firstly, Gregory and Dransfield described the heterogeneity of apoptotic cell-derived EVs in terms of size and content, as well as their mechanism of formation. The authors also discussed how EVs could facilitate intercellular communication in the tumor microenvironment and regulate tumor growth, metastasis, drug resistance, and anti-tumor immunity. However, the importance of EVs generated from apoptotic cells (ApoEVs), in particular from dying tumor cells, in modulating the tumor microenvironment remains to be fully defined. Muhsin-Sharafaldine and McLellan also discussed how ApoEVs generated from tumor cells could exhibit immunosuppressive or immunostimulatory properties depending on the experimental context. In particular, how CD169^+^ macrophages in the lymph node could play a key role in interacting with tumor cell-derived ApoEVs and regulate anti-tumor immunity, as well as how the exposure of phosphatidylserine on tumor cell-derived ApoEVs (e.g., generated after chemo-/radio-therapy) could promote tumor growth through activation of the extrinsic coagulation cascade. It should be noted that in additional to tumor cell-derived ApoEVs, ApoEVs released from a range of untransformed cells during apoptosis could also exhibit immunoregulatory properties. Another review by Caruso & Poon discussed how ApoEVs generated from a range of cell types could modulate immune responses by regulating the efficient clearance of apoptotic cells, antigen presentation, as well as trafficking of cytokines, damage-associated molecular patterns and pathogens.  Caruso and Poon also highlighted the variation in nomenclature and isolation/characterization methods used in a range of ApoEV studies.

## Immunoregulatory Properties of EVs Released From Pathogens

In additional to the importance of EVs in mediating intercellular communication in higher organisms, it is also well-established that a variety of pathogens can release membrane vesicles to modulate host immunity (5), with three research articles in this research topic exploring this area of research. First, Turner et al. examined the mechanism underpinning the entry of Gram-negative bacterial derived outer membrane vesicles (OMVs) into host cells. The authors described the size of OMV being a key determining factor for OMV cargo composition and their host cell entry, with smaller OMVs (20–100 nm) and larger OMVs (90–450 nm) entering host epithelial cells via caveolin-mediated endocytosis and macropinocytosis/endocytosis, respectively. Second, Eichenberger et al. performed proteomic and RNA-seq analysis on parasite (*Nippostrongylus brasiliensis*)-derived EVs (60–160 nm), as well as demonstrated the ability of parasite-derived EVs to suppress inflammation in a murine model of colitis. Lastly, Ofir-Birin et al. described the use of imaging flow cytometry to monitor malaria parasites (*Plasmodium falciparum*)-derived EVs, their uptake into host monocytes, as well as the subsequent translocation of phosphorylated IRF3 into the nucleus in monocytes.

## Afterword

The field of EVs is a rapidly growing area of research, with the identification of new types of EVs, expansion on the cell types or organisms that could release EVs and their functions, as well as the development of novel approaches to study EVs. This research topic has covered a number of cutting-edge discoveries in this field. Lastly, we would like to thank all the authors for their contribution to this research topic and the referees for their prompt and in-depth reviews.

## Author Contributions

All authors listed have made a substantial, direct and intellectual contribution to the work, and approved it for publication.

### Conflict of Interest Statement

The authors declare that the research was conducted in the absence of any commercial or financial relationships that could be construed as a potential conflict of interest.
